# Global insights: ChatGPT's influence on academic and research writing, creativity, and plagiarism policies

**DOI:** 10.3389/frma.2024.1486832

**Published:** 2024-11-08

**Authors:** Muhammad Abid Malik, Amjad Islam Amjad, Sarfraz Aslam, Abdulnaser Fakhrou

**Affiliations:** ^1^Shandong Vocational University of Foreign Affairs, Jinan, China; ^2^Department of School Education, Kasur, Punjab, Pakistan; ^3^Doctoral Studies Department, Faculty of Education and Humanities, UNITAR International University, Petaling Jaya, Malaysia; ^4^Department of Psychological Sciences, College of Education, Qatar University, Doha, Qatar

**Keywords:** artificial intelligence, academic writing, ChatGPT, creativity, plagiarism, policy recommendations

## Abstract

**Introduction:**

The current study explored the influence of Chat Generative Pre-Trained Transformer (ChatGPT) on the concepts, parameters, policies, and practices of creativity and plagiarism in academic and research writing.

**Methods:**

Data were collected from 10 researchers from 10 different countries (Australia, China, the UK, Brazil, Pakistan, Bangladesh, Iran, Nigeria, Trinidad and Tobago, and Turkiye) using semi-structured interviews. NVivo was employed for data analysis.

**Results:**

Based on the responses, five themes about the influence of ChatGPT on academic and research writing were generated, i.e., opportunity, human assistance, thought-provoking, time-saving, and negative attitude. Although the researchers were mostly positive about it, some feared it would degrade their writing skills and lead to plagiarism. Many of them believed that ChatGPT would redefine the concepts, parameters, and practices of creativity and plagiarism.

**Discussion:**

Creativity may no longer be restricted to the ability to write, but also to use ChatGPT or other large language models (LLMs) to write creatively. Some suggested that machine-generated text might be accepted as the new norm; however, using it without proper acknowledgment would be considered plagiarism. The researchers recommended allowing ChatGPT for academic and research writing; however, they strongly advised it to be regulated with limited use and proper acknowledgment.

## 1 Introduction

Artificial intelligence (AI) and chatbots have a long history dating back to the early 1950s when academics first began investigating the concept of AI (Oravec, 2019). ELIZA (created from 1964 to 1966) was the first AI program that attempted to replicate human communication (Pruijt, 2006; Shum et al., 2018). It employed pattern matching and substitution methodologies to generate responses (Weizenbaum, 1966). Newer developments and versions of AI-based chatbots and platforms can be predictive and incorporate emotions (Jungwirth and Haluza, 2023). Over the years, they have evolved for more complex and complicated communications and tasks (King, 2023; Rudolph et al., 2023).

Chat Generative Pre-Trained Transformer (ChatGPT) is one of the first and probably the most popular among AI-based chatbots. It has already had a significant impact in various fields such as medicine, education, creative writing, finance, and reading external data (Baumgartner, 2023; Haenlein and Kaplan, 2019; Lo, 2023; Shidiq, 2023; Zaremba and Demir, 2023). The transformer architecture-based language model was created by OpenAI, which refined it using a vast amount of data (Zhang et al., 2023). It is a complex neural network that processes input sequences using self-attention techniques. These techniques enable it to accommodate input and output sequences and produce text resembling words (Zaremba and Demir, 2023). Due to its advanced AI-based technology, it can produce logical and natural writing in various fields and contexts (Raffel et al., 2020). It has recently been used to answer queries in a comprehensive editorial letter (King, 2023). The model, especially its latest versions, can generate text so close to human writing that it becomes challenging to tell the two apart (Brown et al., 2020). Although amazing and revolutionary, this ability has also created multiple issues such as academic fraud, plagiarism, and degradation of writing skills (Malik, 2024).

### 1.1 Use and impact of ChatGPT on academic and research writing

Due to its transformer design and enormous database, ChatGPT can perform well in various types and levels of writing, such as linguistic knowledge, language processing, information retrieval, summarisation, writing assignments, and research (Huang and Tan, 2023; Hutson, 2022; Shidiq, 2023). It can generate assignments, articles, and dissertations in almost all subjects and research areas (Aljanabi et al., 2023; Salvagno et al., 2023) based on the researcher's ideas, hints, or prompts (Giray, 2023: Shafqat and Amjad, 2024).

Writing with the support of ChatGPT can save time and effort, allowing researchers and scholars to concentrate and spend more time on critical, analytical, and creative aspects (Aljanabi et al., 2023; Malik, 2024). It can also help improve language, grammar, and coherence (Koo, 2023). It can comprehend and respond to complicated cues, making it a valuable writing tool. It can also perform various tasks in research articles and dissertations. Patel and Lam (2023) said that it could help researchers create the first draft of a research article and even make title suggestions. At this stage, ChatGPT-generated work is not always accurate (AlZaabi et al., 2023; Shardlow and Latham, 2023). As a result, the user has to examine and proofread it (Malik, 2024; Nguyen, 2023).

Despite all the benefits and advantages that ChatGPT can bring to academic and research writing, there are some serious concerns, especially about ethical (mostly about academic fraud and unethical use) and cognitive (degradation of cognitive and writing skills due to overreliance on it). The impact of ChatGPT on plagiarism, its policies, and practices will be discussed later; its impact on students' writing skills will be discussed here.

Overreliance on technology has adversely affected writing skills (Ismael et al., 2022). Quite a few studies have feared that ChatGPT will have similar effects on writing skills, especially among students (Hutson, 2022; Shidiq, 2023; Malik, 2024). While acknowledging AI's ability to write academic work, Hutson (2022) also expressed concerns about its negative impact. Shidiq (2023) also feared that “relying too much on ChatGPT can make individuals weak in thinking critically” (p. 354). While talking about the impact of ChatGPT on academic writing, he said, “It is also necessary to realize that not all of these facilities have a good impact on developing several student skills in learning, including creative writing skills” (p. 356). In his study, Malik (2024) also said that overreliance on ChatGPT and other AI tools would degrade students' writing skills.

### 1.2 ChatGPT and creativity

Creativity is the capacity to develop original ideas, emphasize new and novel issues, and solve problems (Boden, 2004). Critical thinking and creativity are essential for modern education (Warner et al., 2021). The introduction of AI has significantly impacted education and creative writing (Shidiq, 2023). It assists teachers in teaching-learning process (Baidoo-Anu and Ansah, 2023) and helps researchers write research (Hutson, 2022).

The rapid influx of ICT in education and online tools redefined the concepts of literacy and creativity (Warner et al., 2021). AI-based chatbots and platforms are again reshaping those concepts and ideas. Ali et al. (2019) said that the students liked to think imaginatively about the use of AI. This makes AI-based education and training imperative for students. Instead of teaching general skills, education systems must encourage students to think critically and creatively using AI. It will help them not only in their academic life but also in their professional career. In their study, Mikalef and Gupta (2021) found a relationship between AI ability and organizational creativity. The study suggested that using AI-based chatbots and platforms improved organizational creativity and productivity.

Oktradiksa et al. (2021) found that AI applications can help students achieve their full potential by emphasizing learning more. ChatGPT can speed up the creation of new ideas and concepts. Open AI claims that ChatGPT- 4 can “generate, edit, and iterate with users on creative and technical writing tasks” (OpenAI, n.d.). Uludag (2023) asked questions to check its ability to generate new ideas. He evaluated its automated responses for creativity and practicality. The findings showed that it was a good source for creative answers. It can also generate texts using reasoning without relying on a direct citation history.

### 1.3 ChatGPT and plagiarism

Plagiarism means using other persons' words, writing, ideas, and concepts without proper acknowledgment and credit (Badke, 2007). Although plagiarism is an old issue that severely threatens research quality, credibility, and integrity, the development of readily available online information and tools in recent decades has made it easier to plagiarize (Malik et al., 2021). This has forced universities to spend substantial funds, time, and efforts to combat it (Gullifer and Tyson, 2010).

ChatGPT has been significantly impacting plagiarism (Malik, 2024). Initial studies have shown that ChatGPT may have been used frequently to generate assignments and research work (Kuhail et al., 2023). Shardlow and Latham (2023) expressed grave concerns about the “inappropriate” use of ChatGPT and other large language models (LLMs), which could “raise concerns about unfair means, plagiarism, and academic misconduct” (p. 2). It also makes it increasingly difficult to tell if a piece of writing is authentic or AI-generated (Dale, 2021). Although quite a few software and tools have been developed to detect ChatGPT and machine-generated text, they are still in their infancy and have shown inconsistent results (Rashidi et al., 2023).

### 1.4 Policies, practices, and policy recommendations about the use of ChatGPT

ChatGPT is still a very new phenomenon in academic and research writing. Not only does “so little policy currently exist” about it (Shardlow and Latham, 2023, p. 54), but the existing policy guidelines and strategies are also quite diverse and even contradictory (Caulfield, 2023). The reactions and responses of universities, research bodies, and regulators vary greatly, from banning it altogether to looking the other way. Even within the same country, different universities, research councils, and regulatory bodies do not appear to be on the same page. In the United Kingdom, for example, 61% of universities did not have any proper policy or guidelines about ChatGPT, 8% banned it completely, 9% banned it unless permitted otherwise, 10% allowed faculty members to decide by themselves, and 12% universities allowed the tool to be used with proper citation unless the faculty instructed otherwise (Caulfield, 2023). This lack of a uniform approach is detrimental and discriminatory, as it gives some students an added advantage while preventing others. This situation calls for a comprehensive, balanced, uniform policy that allows the tool to be used responsibly, equally, and justly.

Many researchers and scholars have called for policy guidelines for its fair and ethical use (Dwivedi et al., 2023; Halaweh, 2023; Malik, 2024; Rahman et al., 2023; Shardlow and Latham, 2023). Malik (2024) called for the development of proper policy and guidelines to ensure “its cautious and careful use, especially by students” (p. 323). Some studies have produced policy recommendations, guidelines, and strategies for it. Shardlow and Latham (2023) published a white paper on its use. Despite their fears and concerns about its misuse, they believed universities and higher education bodies should accept it. However, they suggested various methods and strategies for its fair and ethical use, such as providing guidelines and training, redesigning assessments and evaluations, and using AI-detection software. They also suggested its limited use and proper acknowledgment to avoid unfair and unethical use. Dwivedi et al. (2023) recommended “developing a code of practice for the academic community that offers specific guidelines for using ChatGPT in academic publishing” (p. 56). It also suggested a proper acknowledgment of its use in the article.

There is more consensus among the publication houses than universities and regulatory bodies. Most of the renowned journals are members of the Committee on Publication Ethics (COPE) and follow its guidelines. COPE prohibits AI tools from being listed as one of the authors. It also says that the author(s) should be responsible for the entire text, even if it is generated through AI tools (COPE, 2023). Many publication houses (e.g., Springer, Sage, Springer Nature, Elsevier, Emerald) follow the COPE guidelines by prohibiting authorship or co-authorship by any AI tool or its use for generating or enhancing any image (Elsevier, n.d.; Emerald, 2023; Springer, n.d.; Springer Nature, n.d.). They also ask the authors to mention whether and where they used AI tools.

### 1.5 Research gap and objectives

Like information and communication technology (ICT) and online tools that have changed the concepts and parameters of literacy and creativity (Warner et al., 2021), ChatGPT can influence the concepts and practices of academic and research writing, creativity, and plagiarism. Due to its sudden and exponential growth, many policymakers, regulators, and universities have been caught off guard. There seems to be a lot of confusion and uncertainty due to a lack of uniform and transparent policies and practices about its use (Shardlow and Latham, 2023). It ignites the need to conduct a study that investigates the influence of ChatGPT on academic and research writing, creativity, and plagiarism and develops policy recommendations for its responsible, fair, and ethical use.

This study aims to fill the research gap mentioned above. It explores the perceived impact of ChatGPT on academic and research writing, creativity, and plagiarism. Additionally, it provides recommendations for a viable, balanced, and pragmatic policy for responsible and fair use. More specifically, the study has the following research objectives.

- To investigate the role and influence of ChatGPT in academic and research writing.
- To investigate the influence of ChatGPT on the concept, policies, and practices of creativity in academic and research writing.
- To investigate the influence of ChatGPT on the concept, policies, and practices of plagiarism in academic and research writing.
- To give policy recommendations for the responsible and ethical use of ChatGPT in academic and research writing.

## 2 Research methodology

### 2.1 Research design

This study followed the interpretive research philosophy to explore the researchers' views on the impact of ChatGPT on academic and research writing, creativity, and plagiarism. Interpretivist philosophy focuses on “subjective perspective” and is “more concerned with in-depth variables and factors related to a context” (Alharahsheh and Pius, 2020, p. 41). It believes that “truth and knowledge are subjective, as well as culturally and historically situated, based on people's experiences and their understanding of them” (Ryan, 2018, p. 8). Also, qualitative research (which follows the interpretivism philosophy) is most suitable when the phenomenon is relatively new and lesser known (Patton, 2002). As the phenomena (ChatGPT, its impact on academic and research writing) are relatively less researched, and we wanted perceptions and opinions from the researchers from different cultures and contexts (who may view it differently), an interpretive philosophy-based qualitative research method was chosen for this study.

### 2.2 Participants of the study

Based on the research design, we decided to gather data from the persons (called researchers hereafter) who had done research work on ChatGPT. Researchers were selected using the maximum variation technique. There were five inclusion criteria for them: (a) age must be 35 years or older, (b) must have one or more publications on it or at least be working on AI-related paper(s), (c) have been using it for teaching and/or research purposes for at least one year, (d) affiliated with any university or research center, and (e) belong to different countries.

In the first step, we developed a list of research articles and studies about ChatGPT with the authors' names and email addresses. Various research databases like Google Scholar, Semantic Scholar, ResearhGate, and Science Direct were used to find those studies. The initial list consisted of 37 research articles by 27 different researchers. They belonged to 12 different countries. Later, we contacted them through email addresses to obtain their consent. Twelve researchers from eleven countries initially gave their consent. However, only eight responded from eight different countries (Australia, China, the UK, Brazil, Pakistan, Bangladesh, Iran, and Turkiye) when they were contacted for the second time for interviews. Later, two more researchers were added (one each from Nigeria and Trinidad and Tobago) to include voices from the African Continent and the Caribbean region. Nine of them were males, and one (from Nigeria) was female. Their details are given in Table 1.

**Table 1:** Participants' information.

**Pseudonym**	**Gender**	**Age (years)**	**Qualification**	**Nationality**	**Designation/ position**	**Justification for inclusion**
Ken	Male	44	PhD	UK	Researcher	Using for research for almost two years, two articles on it
Zhu	Male	35	Ph.D. Scholar	China	Lecturer	Using in teaching and research for almost two years, ten articles on it
Sid	Male	50	PhD	Australia	Senior Lecturer	Using in teaching for almost two years, two articles on it
Jose	Male	40	PhD	Brazil	Professor	Using in teaching and research for more than one year, one article on it
Ali	Male	38	PhD	Pakistan	Assistant professor	Using in teaching and research for almost two years, one article on it
Khan	Male	42	PhD	Bangladesh	Associate professor	Using in teaching for almost one year, one article on it
Mosa	Male	50	PhD	Iran	Associate professor	Using in teaching and research for almost two years, one article on it
Alp	Male	45	PhD	Turkiye	Senior lecturer	Using in teaching and research for almost two years, one article on it
Sam	Male	55	PhD	Trinidad and Tobago	Assistant Professor	Using in teaching and research for almost two years, working on ChatGPT-related papers
Laura	Female	36	PhD	Nigeria	Lecturer	Using in teaching and research for over one year, working on ChatGPT-related paper

### 2.3 Data collection tool and technique

Data were collected through semi-structured interviews, which provided room for further investigation (Wilson, 2014). We developed an initial interview guide to explore researchers' perceptions and opinions about the research objectives. It consisted of five main interview questions, i.e., 1. What is the role and influence of ChatGPT on academic and research writing? 2. What has been/will be the influence of ChatGPT on creativity (with a main focus on concepts and parameters, policies, and practices)? 3. What has been/will be the influence of ChatGPT on plagiarism (with a main focus on concepts and parameters, policies, and practices)? 4. What is your university/country's policy about the use of ChatGPT in academic and research writing? 5. What should be the policy for the ethical and responsible use of ChatGPT in academic and research writing? It was later sent to three experts along with the research questions to verify the validity of the content. Supplementary questions were asked when and where necessary.

As the participants belonged to different parts of the world, the interviews were conducted through WhatsApp and Zoom. We took permission from the participants and confirmed the time and date of the interviews. The interviews were recorded as per the research protocol. The ethical protocols of the study were approved by the ethics committee of the School Education Department, Punjab, Pakistan (approval number: 2023-12-31/ED/IRB/013). Informed consent was obtained from all subjects involved in the study.

### 2.4 Data analysis technique

The data were analyzed using the qualitative data analysis software NVivo. Data transcription was carried out using NVivo Transcription. Later, NVivo was used to group each question's responses and generate and connect themes (Jackson and Bazeley, 2019). Braun and Clarke (2006) guide for thematic analysis was used as the methodological foundation for this study. Two of the authors independently examined the findings to review and validate them. The final paper contained themes, keywords, and verbatim quotations.

## 3 Results

The findings were divided into two main parts: the influence of ChatGPT on academic and research writing and current policies and policy recommendations.

### 3.1 Influence of ChatGPT on academic and research writing

NVivo generated seven themes: Opportunity, human assistance, thought-provoking, time-saving, negative attitude, creativity in academic writing, and plagiarism detection. The themes were then categorized into three main categories: the role and influence of ChatGPT on academic and research writing (themes 1 to 5), the impact of ChatGPT on creativity (theme 6), and the impact of ChatGPT on plagiarism, its policies, and practices (theme 7).

#### 3.1.1 Role and influence of ChatGPT on academic and research writing

The first category was about the role and influence of ChatGPT on academic and research writing. It consisted of five themes generated by NVivo: opportunity, human assistance, thought-provoking, time-saving, and negative attitude.

##### 3.1.1.1 Opportunity

The researchers believed that ChatGPT provided multiple new opportunities. It can help students and researchers improve their work and explore more ideas and venues in multiple ways. Ken said, “I just feel the ChatGPT as an enormous opportunity [for students and researchers] to improve their questioning, develop better questions [for the researchers], and get access to a lot of fairly reliable raw material.”

Zhu said that ChatGPT had opened up more opportunities for students and researchers by freeing them from tedious writing tasks. As a result, they will “focus on ideas more,” which could be “a great opportunity for academia.”

##### 3.1.1.2 Human assistance

Many researchers believed that ChatGPT worked as an AI version of human assistants, as it can assist in many ways. Ken commented,

I think it will influence the academic writing skills of researchers in the same way that you know if I had an editorial, a human editorial assistant. Writing in whatever way they imagine, [ChatGPT] will do the same job. For me and the other academics, I was writing papers and turning our ideas into something that is readable and impacts a broader audience.

Other researchers also believed that ChatGPT acted as a human assistant. Khan said it could eliminate the need for an assistant, proofreader, or editor as it can respond according to the instructions.

Jose explained how ChatGPT assisted him in writing and researching work.

I use it almost daily. The main actions are helping develop lesson plans and creating charts and tables; I use them to study and build nutritional plans. I use it to help develop codes for my mathematical software to write LaTEX and Python codes.

Laura said that she employed ChatGPT as a research assistant to get “materials for my academic papers and research writing”. It shows that ChatGPT can be used extensively for multiple tasks and activities. Many other researchers also said they used it for different teaching tasks, such as preparing lesson plans, making charts and tables, and preparing tests and assignments. Many said they used to ask their teaching assistants or secretaries to do those tasks, but ChatGPT had replaced them with much better efficiency and effectiveness.

The researchers admitted they could not trust it completely at that stage and had to go through the tasks. “…but can you trust your teaching assistant blindly? Don't you review and check what they have done? It is the same,” Ali said.

##### 3.1.1.3 Thought-provoking

The researchers also discussed how ChatGPT was helping them explore new ideas and concepts. Jose expressed how it helped him explore new horizons. He shared his experience in these words:

I discovered it in December 2022 and immediately tried to test it as a simple search engine. 'How many years does the pedagogy course last in Brazil?' and 'Possibilities to do an exchange program for the exterior.' These were the questions that I initially thought of. Later, while writing some scientific articles, I used the tool to find international references on some theoretical perspectives. I remember that the answers I got to this simple question made me think about how education can be affected by this type of resource.

Ken also said that initially, the potential of ChatGPT was not fully realized, but later, he understood that it was much more than an AI-based proofreader or even a writer. He explained how his interaction with ChatGPT helped him explore new ideas.

The first time I used it, I thought it was a toy, so I played with it and got it to write ridiculous things. However, soon, I found that some of the responses it was generating were very thought-provoking, and I realized that there was an opportunity here to have some conversations that were very, very thought-provoking. Then, after a week or two, I thought people would use this professionally. Now, schools, colleges, and universities must help students learn to use it to improve their performance.

Ken believed that people would soon use it to solve their professional tasks. He also emphasized that educational institutions should start helping students learn how to use it productively and effectively.

ChatGPT combines most of the human knowledge that has been put onto the internet and presents it in a sensible, logical, and human-like manner. Thus, interaction with it gives knowledge from different perspectives. Khan described it beautifully, saying it “felt like talking to different experts simultaneously.”

##### 3.1.1.4 Time-saving

The researchers believed that ChatGPT could help students, researchers, and scholars save much time on different tasks. Jose said, “I found myself thinking about how much time can be saved in scientific production or even in my tasks as a teacher.” Zhu said that ChatGPT did different tasks, which could help “save time for the researchers.” Sid also said that it “saves much time.” Mosa said, “I have been experimenting with ChatGPT in Iran for translation purposes for my research, which helps save time.”

ChatGPT can also help teachers prepare materials and answer students' questions. Khan recalled his experiences with it: “I have used ChatGPT daily to find answers related to my daily class topics. It gives me quick answers. So, it saves time, and I can do other tasks in the remaining time.” Ken said, “Sometimes when I had to answer students' questions, I took help from ChatGPT, which helped me save time. Otherwise, I had to look for different books and other resources.”

##### 3.1.1.5 Negative attitude

ChatGPT is a relatively new platform with much potential; however, there are also many reservations and concerns about it. Sid expressed some of these fears by saying, “Students will forget how to write due to their overreliance on it.” Sam thought that the “tool is not understood, and it is misused”. He thought it could “make the human mind artificial if it depends too heavily on ChatGPT.”

Some of the other researchers expressed similar concerns. They believed it might eliminate or at least decrease the importance of writing skills as it could write everything for everyone. Ali thought that as calculators reduced the importance of learning mathematical calculations, ChatGPT may reduce the focus and attention on writing skills.

#### 3.1.2 Impact of ChatGPT on creativity

The second category (based on the sixth theme: creativity in academic writing) was about the impact of ChatGPT on creativity, especially in academic and research writing. The researchers were asked different questions about the impact of ChatGPT on creativity. They were divided on this topic. Many argued that it would modify the concepts of creativity as online tools and platforms had done earlier. Zhu said,

It will drastically change the concept and the skills for creativity. See how online tools have changed what are considered creative skills. The whole concept of creativity has changed due to digitalization. Artificial intelligence is going to have an even greater impact.

Ali also believed that ChatGPT would “modify creativity and essential skills.” Explaining his point by giving an example of statistical software, he said

Before statistical software like SPSS, people had to learn statistical formulas to be creative and do creative work. Now, how many, how many people know them? They just use the software. They put the data in it, press some buttons, and complete the task. So they need different kinds of skills to be creative.

Khan suggested that “creative writing will no longer be the ability to write independently, but utilizing ChatGPT to write independently.”

On the other hand, some thought that ChatGPT would not change the concepts and parameters of creativity. They argued that it was “just [a] helping tool” (Ken). Alp further explained it in these words.

[It is like] asking an assistant to write a letter, directing her what to write, and then looking at it for corrections. Can we say that a person is no longer creative or has lost creative skills in writing because he asked someone to do writing?

Alp said that despite the emergence of ChatGPT, the role and importance of creativity in academic writing would not diminish. He stated that only those who were good at academic writing could produce good writing with it. Sid also supported it, saying,

I do not believe that the level and concept of creativity will change. I think that with new tools, new forms of use arise. The action of researching, writing, and teaching will be re-assigned, but this does not necessarily imply that we will be more or less creative. This is a more intrinsic concept that depends on other variables.

It is pretty clear that the researchers' views about the influence of ChatGPT on the concepts and parameters of creativity are divided. It may be because ChatGPT is a new platform that is still relatively untested. Its impact and influence can be adequately felt only after it has been used widely and substantially.

We also realized that the researchers' views about the impact of ChatGPT on creativity were somewhat linked to their conceptualization of creativity. Those who viewed creativity as purely human characteristic were more inclined to believe that it would not affect the concepts and practices of creativity; however, those who viewed tools as part of the creativity were more inclined to opine that it would.

#### 3.1.3 Impact of ChatGPT on plagiarism, its policies, and practices

This category mainly investigated the impact of ChatGPT on plagiarism, its policies, and practices. An overwhelming majority of the researchers expressed concerns about how ChatGPT was already employed for plagiarism and academic fraud. Sam shared how it was already being used to produce plagiarized content in secondary schools in Trinidad and Tobago. He said, “Many students aged 15-17 when they have to prepare for their final term exams, have a school-based assessment where they have to produce something original, say poems. Quite a few of them use [ChatGPT] to write.”

Many researchers believed that ChatGPT would slowly change the concepts of plagiarism over time. Ali said that ChatGPT was initially viewed negatively as many people thought it would not only degrade human skills and abilities (making them machine-dependent) but also lead to a rise in plagiarism. Jose also feared the same, saying, “Plagiarism as appropriation of an idea or concept, or even pure copying of generated answers, will be the main difficulties of the academic community, especially when ChatGPT increases its robustness.” He said that it would greatly influence the policies and practices about plagiarism. “In my opinion, publishers, scientific conferences, and even institutions as producers of scientific content will change their data production and plagiarism policies, considering the power of this tool.” Khan also stated that “many people in education see it as a threat to academic integrity.”

On the other hand, some thought that ChatGPT was the way forward. Sid said that it was like using a calculator or Grammarly. He said,

How many people think that one has indulged in academic theft because they use a calculator for mathematical calculation or Grammarly for proofreading? The only thing they should mention in the methodology section is whether ChatGPT was used or not.

He said we could not stick to the old concepts and parameters with the changing times and technologies. The role and use of ChatGPT would continue to increase as the chatbot would further increase its “efficiency and performance.” Ken also agreed that academics should regulate it or develop protocols for its ethical and responsible use rather than outright banning it or even seeing it as a threat. He suggested that it should be mentioned clearly in the research article where and how much ChatGPT was used, thus clarifying things and “giving credit to the assistant.”

It is important to note that in many cases, the researchers' thoughts about the impact of ChatGPT on the parameters and policies pertaining to plagiarism were opinions-based. The causes may lie behind the fact that ChatGPT and other AI-based chatbots and tools are still relatively new phenomena and have not been explored deeply and multi-dimensionally. Furthermore, in many cases, especially when it comes to universities and other educational institutions, policies and practices about ChatGPT are unclear and contradictory, which may also contribute to contrasting opinions and viewpoints.

### 3.2 Policies and policy recommendations for the use of ChatGPT

Finally, the researchers were asked about current policies on using ChatGPT in their universities and policy recommendations for it. Table 2 contains the crux of their responses.

**Table 2:** Current policies on ChatGPT and policy recommendations.

**Pseudonym**	**Policy in the university (as of November 2023)**	**Policy recommendation**
Ken	There are no clear guidelines; it depends on the faculty member	Regulate. Use for specific purposes only. We must acknowledge it properly.
Zhu	No clear policy	Allow for faculty members Ban for students due to fears of academic fraud and hindering their learning.
Sid	Allowed, but one must acknowledge it in the work	Regulate. Limited use (< 20%) with acknowledgment.
Jose	There are no clear guidelines; however, software is used for AI detection. If the percentage is too high, it may be considered plagiarized.	Regulate. Limited use with acknowledgment
Ali	No clear policy. Initially, the university flagged the thesis if AI detection was 5% or more; however, later, this practice was discontinued.	Regulate. With proper acknowledgment, allow for not more than 19% of text to be AI-generated.
Khan	Banned over the fears of unethical use and plagiarism	Allow for the faculty and may be for post-graduate and PhD students. Ban for undergraduate students.
Mosa	It is officially banned in Iran; however, quite a few university students and faculty members use it with the help of a VPN.	Regulate. Limited use (like similarity index, < 20%) with acknowledgment
Alp	Not allowed	Regulate. Allowed within a specific limit with acknowledgment
Sam	No clear policy	Regulate. Train about its ethical and proper use of acknowledgment.
Laura	No clear policy	Regulate. Less strictly for the faculty, more strictly for the students

Regarding current policies in their universities, six researchers (Ken, Zhu, Jose, Ali, Sam, and Laura) said that there was no clear policy about ChatGPT. Ken said that his university had left the matter in the hands of the faculty members. He explained the rationale: “You know the nature of the subject, the type of assignments, and so many other factors. Faculty members can decide when and where to allow it. They have autonomy.” Zhu also said that the university had no uniform policy; however, different schools had different practices.

Ali said that initially, his university would check the thesis for AI-generated text, and if it were 5% or higher, it would be returned to students for corrections. Later, the university discontinued this practice, which resulted in no checks. However, individual teachers would set their own guidelines for assignments and project reports. Jose also said that many departments checked the dissertations and that they would be returned if the percentage was too high; however, there was no uniform policy.

Khan, Mosa, and Alp said that ChatGPT was prohibited in their universities. In some cases (Khan and Alp), the university banned it due to fears of academic fraud and unethical use; in others (Mosa), it was completely banned in the country.

On the other hand, Sid said that it was allowed for academic and research purposes; however, it had to be acknowledged properly. As a result, it was more of a regulated use than a blanket permission.

When it came to policy recommendations, there was more consensus. The researchers emphasized four key points: permission, regulation, acknowledgment, and limited use. All of them believed that ChatGPT was here to stay. Sid said, “You cannot turn your back to it; you cannot turn a blind eye. ChatGPT and artificial intelligence are a reality. They are the future.” Sam also echoed the same. He said, “ChatGPT is part of the future. We have to deal with it”.

The researchers believed that rather than living in a world of denial and trying to ban it, it would be wiser to regulate it. Ali said, “Policy studies have shown that banning something does not work often, especially as important and significant as artificial intelligence. It is like computers. Their use will only increase over time.” Both Mosa and Alp mentioned how the ban on ChatGPT did not work in their universities. “This is a world of fast-evolving technology. You cannot stop people. You cannot censor things. Everyone has access to VPNs. They will find ways”. According to them, the best option was to have proper standards and protocols for its fair and ethical use. Ken said, “There should be policies and protocols to regulate it, to ensure that it is used properly and ethically.” Many researchers also emphasized that these regulations should be universal and uniform so that one group does not get any added advantage over the others. Ali said,

Like [for] plagiarism, we have the APA manual. Most universities worldwide use it as guidelines for education, psychology, and other similar disciplines. No matter where you come from, you follow similar protocols. We need to have standardized policies and regulations for ChatGPT. Otherwise, imagine that one university allows it and the other does not. Don't you think it will create disparities?

All researchers believed ChatGPT should be acknowledged appropriately to ensure transparency and research ethics. Zhu said, “One should know when, where, and how much ChatGPT is used. It should be clear and transparent.”

Khan and Zhu suggested that faculty should be allowed to use ChatGPT while students may not, as it could interfere with their learning. They were also afraid that the students might go for “the easy way” and “rely upon it way too much” (Zhu). However, Khan thought post-graduate students might be allowed to use it as they were “mature and sensible enough.” Although Laura did not think it should be banned for students, she believed there should be stricter regulations for them as they were more prone to plagiarism.

Many researchers also suggested providing proper awareness campaigns and training for its ethical, fair, and responsible use. Sam emphasized “retraining” as old training was inadequate to meet the challenges of this artificial intelligence age.

Although many researchers recommended limited use of ChatGPT, three of them (Sid, Ali, and Mosa) suggested a specific limit (19% or below). Ali said: “Not more than 19%, just like the similarity index. I think that would be sensible. One can use AI-generated text to some limit, but not overly rely on it. He further added that ”even that should be with proper acknowledgment.”

## 4 Discussion

ChatGPT has quickly created many ripples in academic circles (Baumgartner, 2023; Lo, 2023; Malik, 2024). Within a few months of its release, it attracted more than a hundred million people who used it for various purposes (Kurian et al., 2023). Multiple studies have discussed its immense influence in different fields (Haenlein and Kaplan, 2019; Lo, 2023; Shidiq, 2023; Zaremba and Demir, 2023). The current study was designed to explore its role and impact on academic and research writing, concepts, parameters, and practices of creativity and plagiarism. Moreover, it presents policy recommendations for its responsible, fair, and ethical use.

The findings highlighted the role, importance, and potential of ChatGPT and how it can influence academic and research writing. The researchers saw ChatGPT as an opportunity that would open many new windows for students, teachers, scholars, and researchers. Many studies have realized this potential (Kuhail et al., 2023; Lo, 2023).

The researchers believed ChatGPT would provide more opportunities by helping academics, researchers, and students improve their concepts, explore new ideas, and solve academic issues. It can also assist in writing abstracts and other academic and research work. Hutson (2022) also suggested that ChatGPT can help scholars with abstracts, literature reviews, summaries, text organizations, citation styles, and title ideas. In this way, it can help save time and effort, which can be used for more creative and productive things. Aljanabi et al. (2023) said that its most valuable contribution would be to work as an assistant to save time and effort. AlZaabi et al. (2023) also suggested that it could relieve some of the burden and pressure on the researchers. This study predicts that ChatGPT will change academic writing, work habits, and styles.

Being thought-provoking was another quality that the researchers pointed out. Unlike regular search engines that collect only information, ChatGPT can suggest, synthesize, and develop new concepts and ideas. Uludag (2023) said that it could contribute significantly to the exponential development of different fields due to its ability to lead to new concepts and ideas. It can also help students and scholars by providing valuable feedback on their scholarly work. Nguyen (2023) also pointed out its ability to assist with grammar, language, and feedback. Its ability to understand and react to nuanced inputs further enhances its utility as a writing and learning tool. It can help students comprehend and summarize complex materials and generate essay ideas.

Despite the advantages mentioned above, some researchers had reservations and a negative attitude toward it. They argued that ChatGPT also had side effects and negative influences. They feared that it would degrade their writing skills. Studies have shown that too much reliance on technology degrades writing skills (Ismael et al., 2022). In his study, Malik (2024) expressed similar concerns about ChatGPT. Shidiq (2023) also feared that ChatGPT could negatively affect some essential skills, such as writing. An experimental study by Niloy et al. (2024) also found a negative association between ChatGPT and students' academic writing skills. The researchers also questioned the authenticity and accuracy of ChatGPT-generated text at this stage. Some studies have also raised these concerns (AlZaabi et al., 2023; Shardlow and Latham, 2023).

The second key research question was its influence on creativity concepts, parameters, and practices. The researchers were divided on this issue, as some believed it would modify the concept and parameters of creativity: what defines creativity and what skills are considered essential. The influx of technology has already influenced the concepts and parameters of literacy, learning, and creativity (Amin et al., 2021). Friedman (2007) said that before the advent and widespread use of online tools and technology, critical skills were undertaking and following orders, teamwork, honesty, and being efficient; however, in the technology-infused current era, the emphasis has been shifted to deeper and more extensive critical thinking and the ability to use modern technologies effectively. The researchers believe that in the coming days, the required skills may not be the ability to write effectively but to use ChatGPT for it. Writing may be outsourced to these platforms, and the ability and skills to utilize them effectively would replace actual writing. Other researchers disagreed with it. They believed that since creativity was the ability to generate unique ideas, highlight fresh and unusual concerns, and improve problem-solving (Boden, 2004), ChatGPT would only help utilize those skills more effectively. According to them, ChatGPT would be more of a human assistant than an independently thinking mind that can be outsourced for a task. Muhammad et al. (2023) also argued that despite all the technological advancements, “these are the people behind the machines who matter” (p. 460). We also believe that lower-level tasks and write-ups may be outsourced to ChatGPT; however, when it comes to more complex tasks with innovative thinking, human minds would still prevail (at least at this stage).

The advent of modern technology, such as online and digital tools, has played a dual role in plagiarism (Malik et al., 2021). On the one hand, they help detect plagiarism through various software and programs; on the other, they make plagiarism and avoiding plagiarism detection relatively easy (Chang et al., 2015; Malik et al., 2021). When talking about the impact of ChatGPT on plagiarism, many of them feared that it would increase plagiarism in academic writing due to its ease of use and ability to generate human-like text. Many studies have raised this concern (Dale, 2021; Malik, 2024; Shardlow and Latham, 2023). However, the researchers said that ChatGPT-generated text was not undetectable. Some pointed out that different software had already started incorporating options to detect machine-generated text. They thought that with the advancements in AI detection software, it may be easier to detect such practices.

Another key question was about the impact of ChatGPT on concepts, parameters, and policies about plagiarism. The researchers expected ChatGPT to influence and change the concepts, parameters, and practices of plagiarism, as statistical software had done earlier. They pointed out that in almost every case, the person doing the statistical analysis would ask the program to do it. In many cases, they would not even know the formula. They highlighted the fact that outsourcing the analysis to software was not plagiarism. The only thing was that it had to be appropriately mentioned in the work. They suggested that a time may come when writing would also be outsourced to ChatGPT. According to them, that would not be plagiarism but a new way of writing. Kuhail et al. (2023) also commented that the notions of attribution and original work would be questioned in a world where persuasive chatbots were widely available. This may lead to redefining plagiarism, its parameters, and practices. Plagiarism may not be defined as an act where machine-generated text is used but where it is not properly mentioned and acknowledged.

When asked about the current policies about ChatGPT in their universities, they revealed that there was no uniform policy. Different universities in different countries (and even different departments within the same university) had different policies and practices. Previous studies have also discussed the lack of uniform and standardized policies and regulations on the use of ChatGPT and other AI tools (Caulfield, 2023; Dwivedi et al., 2023; Halaweh, 2023; Malik, 2024; Rahman et al., 2023; Shardlow and Latham, 2023). The researchers questioned this approach, fearing such practices would lead to confusion, malpractice, and disparities. They suggested standardized policies and protocols for the just, equal, and fair use of ChatGPT and other AI tools.

When asked about the policy recommendations, the researchers unanimously opposed banning ChatGPT. Pearson (2023) also said that ChatGPT was here to stay. Instead of banning or disregarding it, they advocated to regulate it. Some had reservations about its use by undergraduate students; however, they thought that faculty members and post-graduate students should be allowed to use it. They suggested limited use of ChatGPT, with some suggesting a cap of 19%, just like the similarity index. They also recommended its proper acknowledgment in the paper. COPE also suggests the same in research publications (COPE, 2023).

## 5 Policy recommendations

We believe that ChatGPT is here to stay. Turning a blind eye or banning it altogether may only be counterproductive. Those countries and universities banning ChatGPT are not preparing their students for an AI-based future. We also suggest that the policies and regulations should be universal and uniform (or at least have some minimum universal guidelines). Otherwise, they will create disparities where one group can gain an advantage while the other is not.

Based on the literature review and the study findings, we give the following policy recommendations for its fair, responsible, and ethical use in academic and research writing.

- There should be a proper acknowledgment of the use of ChatGPT. It should be mentioned clearly either in methodology or in acknowledgment. Its use without proper acknowledgment should be considered plagiarism and prohibited.
- Its use should be limited, and ChatGPT-generated text may not exceed 19% of the total text.
- If the ChatGPT-generated text is more than 19% of the text, it should be checked by another software. If the second software also shows more than 19% detection, it should be returned for revision. However, it may be accepted if the second software shows 19% or less.
- Regular workshops and seminars should held and focus on its responsible, ethical, and just use. Both teachers and students should be informed about it.
- Ethics and protocols for responsible, ethical, and just use of ChatGPT should be included in relevant courses (e.g., academic writing, research methodologies, etc.).
- At the undergraduate level, ChatGPT may not be allowed initially; however, it may be allowed once there is sufficient education and training on its ethical and responsible use.

It is important to note that these policy recommendations are more of basic guidelines that may be applicable in many cases. Every country's context, infrastructure, mindset, socioeconomic background, and education level are different. Furthermore, universities are usually autonomous bodies with their system, culture, and background. As a result, countries and universities may formulate more specific policies based on their culture, context, and background; however, as said earlier, these policy recommendations can serve as the starting point (if not as the basic framework).

## 6 Conclusions

This study explored the influence of ChatGPT on academic and research writing with a particular focus on creativity and plagiarism. The researchers pointed out many positive influences, such as providing more opportunities, working as a human assistant, leading to new and innovative ideas, and saving time; however, some also feared that it could degrade academic and research writing and increase plagiarism. Many argued that it would change the parameters and practices of creativity and plagiarism. Creativity may no longer be limited to creatively independent writing but collaborating with ChatGPT and other AI tools. In the same way, its use for academic and research writing may be accepted as the new norm; however, doing it without proper acknowledgment may be considered plagiarism.

Later, we presented policy recommendations for its ethical, responsible, and fair use. We recommend that it be regulated and initially allowed for faculty members and post-graduate students. However, its use may be restricted to 19% or below with proper acknowledgment. Students and teachers must be educated and trained about its ethical and responsible use. Even undergraduate students may be allowed to do so once proper awareness and understanding are developed.

Whereas COPE and major publishing houses have already developed relatively efficient and useful protocols for using ChatGPT, the same cannot be said about higher education institutions. This study has tried to fill this research gap. However, despite being a relatively new phenomenon, ChatGPT and AI-based chatbots and platforms are evolving at an unprecedented pace. Consequently, any policies or regulations about them must be revised occasionally based on the latest developments. We also understand that every country's context, academic and research culture, infrastructure, and socioeconomic background are different, necessitating different policies depending on the criteria above. However, these policy recommendations can serve as a basic framework for developing more specific policies for every country. As a result, this study will have long-term consequences and implications.

## Data Availability

The datasets presented in this article are not readily available because the dataset contains sensitive personal information disclosed during the interviews. Access to the dataset is restricted to protect the confidentiality and identity of the participants. Requests to access the datasets should be directed to amjad_14@yahoo.com.

## References

[B1] Alharahsheh H. H. Pius A. (2020). A review of key paradigms: positivism VS interpretivism. Global Acad. J. Humanit. Soc. Sci. 2, 39–43.

[B2] Ali S. Payne B. H. Williams R. Park H. W. Breazeal C. (2019). “Constructionism, ethics, and creativity: developing primary and middle school artificial intelligence education,” in International Workshop on Education in Artificial Intelligence K-12 2 (Palo Alto, CA), 1–4.

[B3] Aljanabi M. Ghazi M. Ali A. H. Abed S. A. (2023). ChatGpt: open possibilities. Iraqi J. Comp. Sci. Mathem. 4, 62–64. 10.52866/20ijcsm.2023.01.01.0018

[B4] AlZaabi A. ALamri A. Albalushi H. Aljabri R. AalAbdulsallam A. (2023). ChatGPT applications in academic research: a review of benefits, concerns, and recommendations. Biorxiv 2023, 1–23. 10.1101/2023.08.17.553688

[B5] Amin H. Malik M. A. Akkaya B. (2021). Development and validation of digital literacy scale (DLS) and its implication for higher education. Int. J. Dist. Educ. E-Learn. 7, 24–43. 10.36261/ijdeel.v7i1.2224

[B6] Badke W. (2007). Give Plagiarism the Weight It Deserves, 58–60. Available at: https://www.researchgate.net/publication/292790712_Give_plagiarism_the_weight_it_deserves (accessed May 5, 2024).

[B7] Baidoo-Anu D. Ansah L. O. (2023). Education in the era of generative artificial intelligence (AI): Understanding the potential benefits of ChatGPT in promoting teaching and learning. J. AI 7, 52–62. 10.61969/jai.1337500

[B8] Baumgartner C. (2023). The potential impact of ChatGPT in clinical and translational medicine. Clin. Transl. Med. 13, 1–4. 10.1002/ctm2.1206 36854881 PMC9974599

[B9] Boden M. A. (2004). The Creative Mind: Myths and Mechanisms. London: Psychology Press.

[B10] Braun V. Clarke V. (2006). Using thematic analysis in psychology. Qual. Res. Psychol. 3, 77–101. 10.1191/1478088706qp063oa32100154

[B11] Brown T. Mann B. Ryder N. Subbiah M. Kaplan J. D. Dhariwal P.. (2020). Language models are few-shot learners. Adv. Neural Inf. Process. Syst. 33, 1877–1901.

[B12] Caulfield J. (2023). University Policies on AI Writing Tools | Overview & List. Amsterdam: Scribbr. Available at: https://www.scribbr.co.uk/using-ai-tools/chatgpt-university-policies-uk/ (accessed March 7, 2024).

[B13] Chang C. M. Chen Y. L. Huang Y. Y. Chou C. (2015). Why do they become potential cyber-plagiarisers? Exploring the alternative thinking of copy-and-paste youth in Taiwan. Comput. Educ. 87, 357–367. 10.1016/j.compedu.2015.07.006

[B14] COPE (2023). Authorship and AI Tools. The Committee on Publication Ethics (COPE). Available at: https://publicationethics.org/cope-position-statements/ai-author (accessed July 5, 2024).

[B15] Dale R. (2021). GPT-3: What's it good for? Nat. Lang. Eng. 27, 113–118. 10.1017/S1351324920000601

[B16] Dwivedi Y. K. Kshetri N. Hughes L. Slade E. L. Jeyaraj A. Kar A. K.. (2023). “So what if ChatGPT wrote it?” Multidisciplinary perspectives on opportunities, challenges and implications of generative conversational AI for research, practice and policy. Int. J. Inform. Managem. 71:102642. 10.1016/j.ijinfomgt.2023.102642

[B17] Elsevier (n.d.). Publishing Ethics. London: Elsevier. Available at: https://www.elsevier.com/about/policies-and-standards/publishing-ethics#0-publishing-ethics (accessed 2024).

[B18] Emerald (2023). Emerald Publishing's Stance on AI Tools and Authorship. Leeds: Emerald. Available at: https://www.emeraldgrouppublishing.com/news-and-press-releases/emerald-publishings-stance-ai-tools-and-authorship#:~:text=Secondly%2C%20any%20use%20of%20AI,policies%20come%20into%20effect%20immediately (accessed May 5, 2024).

[B19] Friedman T. L. (2007). The World is Flat: A Brief History of the Twenty-First Century. New York: Farrar, Straus and Giroux.

[B20] Giray L. (2023). Prompt engineering with ChatGPT: a guide for academic writers. Ann. Biomed. Eng. 51, 2629–2633. 10.1007/s10439-023-03272-4 37284994

[B21] Gullifer J. Tyson G. A. (2010). Exploring university students' perceptions of plagiarism: A focus group study. Stud. Higher Educ. 35, 463–481. 10.1080/03075070903096508

[B22] Haenlein M. Kaplan A. (2019). A brief history of artificial intelligence: on the past, present, and future of artificial intelligence. Calif. Manage. Rev. 61, 5–14. 10.1177/0008125619864925

[B23] Halaweh M. (2023). ChatGPT in education: strategies for responsible implementation. Contemp. Educ. Technol. 15:ep421. 10.30935/cedtech/13036

[B24] Huang J. Tan M. (2023). The role of ChatGPT in scientific communication: writing better scientific review articles. Am. J. Cancer Res. 13:1148. 37168339 PMC10164801

[B25] Hutson M. (2022). Could AI help you to write your next paper? Nature 611, 192–193. 10.1038/d41586-022-03479-w 36316468

[B26] Ismael K. O. Saeed K. A. Ibrahim A. S. Fatah D. S. (2022). Effects of auto-correction on students' writing skill at three different universities in Sulaimaneyah City. Arab World Engl. J. 8, 231–245. 10.24093/awej/call8.16

[B27] Jackson K. Bazeley P. (2019). Qualitative Data Analysis with NVivo. Thousand Oaks, CA: Sage.

[B28] Jungwirth D. Haluza D. (2023). Artificial Intelligence and the Sustainable Development Goals: GPT-3s Reflections on the Society Domain. 10.20944/preprints202303.0025.v1

[B29] King M. R. (2023). The future of AI in medicine: a perspective from a chatbot. Ann. Biomed. Eng. 51, 291–295. 10.1007/s10439-022-03121-w 36572824

[B30] Koo M. (2023). The importance of proper use of ChatGPT in medical writing. Radiology 307, e230312. 10.1148/radiol.230312 36880946

[B31] Kuhail M. A. Alturki N. Alramlawi S. Alhejori K. (2023). Interacting with educational chatbots: a systematic review. Educ. Inform. Technol. 28, 973–1018. 10.1007/s10639-022-11177-3

[B32] Kurian N. Cherian J. M. Sudharson N. A. Varghese K. G. Wadhwa S. (2023). AI is now everywhere. Br. Dent. J. 234, 72. 10.1038/s41415-023-5461-1 36707552

[B33] Lo C. K. (2023). What is the impact of ChatGPT on education? A rapid review of the literature. Educ. Sci. 13:410. 10.3390/educsci13040410

[B34] Malik M. A. (2024). Challenges and opportunities about ChatGPT in higher education: a qualitative study about university teachers in Pakistan. Voyage J. Educ. Stud. 4, 315–324. 10.58622/vjes.v4i2.166

[B35] Malik M. A. Mahroof A. Ashraf M. A. (2021). Online university students' perceptions on the awareness of, reasons for, and solutions to plagiarism in higher education: the development of the AS&P Model to combat plagiarism. Appl. Sci. 11:12055. 10.3390/app112412055

[B36] Mikalef P. Gupta M. (2021). Artificial intelligence capability: Conceptualisation, measurement calibration, and empirical study on its impact on organisational creativity and firm performance. Inform. Managem. 58, 1–20. 10.1016/j.im.2021.103434

[B37] Muhammad A. Malik M. A. Malik H. A. M. (2023). Inculcating ethical and moral values amongst the e-learners: Proposing a model for e-learning platforms. *Eur. J. Educ. Res*. 12, 455–465. 10.12973/eu-jer.12.1.455

[B38] Nguyen M. H. (2023). Academic writing and AI: Day-1 experiment. Center for Open Sci. 2023. 10.31219/osf.io/xgqu5

[B39] Niloy A. C. Akter S. Sultana N. Sultana J. Rahman S. I. U. (2024). Is Chatgpt a menace for creative writing ability? An experiment. J. Comp. Assist. Learn. 40, 919–930. 10.1111/jcal.12929

[B40] Oktradiksa A. Bhakti C. P. Kurniawan S. J. Rahman F. A. (2021). Utilisation artificial intelligence to improve creativity skills in society 5.0. J. Phys.: Conf. Series 1760, 1–6. 10.1088/1742-6596/1760/1/012032

[B41] OpenAI (n.d.). GPT-4 Can Solve Difficult Problems With Greater Accuracy, Thanks to its Broader General Knowledge and Problem Solving Abilities. San Francisco, CA: Open AI. Available at: https://openai.com/gpt4 (accessed 2024).

[B42] Oravec J. A. (2019). Artificial intelligence, automation, and social welfare: some ethical and historical perspectives on technological overstatement and hyperbole. Ethics Soc. Welfare 13, 18–32. 10.1080/17496535.2018.1512142

[B43] Patel S. B. Lam K. (2023). ChatGPT: the future of discharge summaries? Lancet Digit. Health 5, 107–108. 10.1016/S2589-7500(23)00021-3 36754724

[B44] Patton M. Q. (2002). Qualitative Research and Evaluation Methods. London: Sage.

[B45] Pearson T. (2023). AI-like it or not, it is here to stay. ADCES in Pract. 11, 6–7. 10.1177/2633559X231191659

[B46] Pruijt H. (2006). Social interaction with computers: an interpretation of Weizenbaum's ELIZA and her heritage. Soc. Sci. Comput. Rev. 24, 516–523. 10.1177/0894439306287247

[B47] Raffel C. Shazeer N. Roberts A. Lee K. Narang S. Matena M.. (2020). Exploring the limits of transfer learning with a unified text-to-text transformer. J. Mach. Learn. Res. 21, 5485–5551.

[B48] Rahman M. M. Terano H. J. Rahman M. N. Salamzadeh A. Rahaman M. S. (2023). ChatGPT and academic research: a review and recommendations based on practical examples. J. Educ. Managem. Dev. Stud. 3, 1–12. 10.52631/jemds.v3i1.175

[B49] Rashidi H. H. Fennell B. D. Albahra S. Hu B. Gorbett T. (2023). The ChatGPT conundrum: human-generated scientific manuscripts misidentified as AI creations by AI text detection tool. J. Pathol. Inform. 14, 100342. 10.1016/j.jpi.2023.100342 38116171 PMC10727991

[B50] Rudolph J. Tan S. Tan S. (2023). ChatGPT: Bullshit spewer or the end of traditional assessments in higher education? J. Appl. Learn. Teach. 6, 1–22. 10.37074/jalt.2023.6.1.9

[B51] Ryan G. (2018). Introduction to positivism, interpretivism and critical theory. Nurse Res. 25, 41–49. 10.7748/nr.2018.e1562 29546962

[B52] Salvagno M. Taccone F. S. Gerli A. G. (2023). Can artificial intelligence help with scientific writing? Critical Care 27, 1–5. 10.1186/s13054-023-04380-2 36841840 PMC9960412

[B53] Shafqat F. Amjad A. I. (2024). Examining students' perceptions, experiences, and ethical concerns about using ChatGPT for academic support: a phenomenological study. Pakist. Soc. Sci. Rev. 8, 443–455. 10.35484/pssr.2024(8-II)36

[B54] Shardlow M. Latham A. (2023). “ChatGPT in computing education: a policy whitepaper,” in Discussion Paper. Council of Professors and Heads of Computing, UK. Available at: https://e-space.mmu.ac.uk/633469/

[B55] Shidiq M. (2023). “The use of artificial intelligence-based Chat-GPT and its challenges for the world of education; from the viewpoint of the development of creative writing skills,” in Proceeding of International Conference on Education, Society, and Humanity 1, 360–364.

[B56] Shum H. Y. He X. D. Li D. (2018). From Eliza to XiaoIce: challenges and opportunities with social chatbots. Front. Inform. Technol. Electr. Eng. 19, 10–26. 10.1631/FITEE.1700826

[B57] Springer (n.d.). Artificial Intelligence (AI). Cham: Springer. Available at: https://www.springer.com/gp/editorial-policies/artificial-intelligence–ai-/2542850 (accessed June 6, 2024).

[B58] Springer Nature (n.d.) Editorial Policies. Cham: Springer Nature. Available at: https://www.springernature.com/gp/policies/editorial-policies (accessed June 6 2024).

[B59] Uludag K. (2023). Testing Creativity of ChatGPT in Psychology: Interview with ChatGPT. Available at: https://ssrn.com/abstract=4390872

[B60] Warner S. C. Malik M. A. Mohammed J. H. (2021). ICT professional development workshops and classroom implementation challenges: perceptions of secondary school teachers in Trinidad and Tobago. Int. J. Innovat. Teach. Learn. 7, 1–19. 10.35993/ijitl.v7i1.1507

[B61] Weizenbaum J. (1966). A computer program for the study of natural language communication between man and machine. Commun. ACM 9, 36–45. 10.1145/365153.365168

[B62] Wilson C. (2014). Semi-structured interviews. Interv. Techniq. UX Practit. 1, 23–41. 10.1016/B978-0-12-410393-1.00002-8

[B63] Zaremba A. Demir E. (2023). ChatGPT: Unlocking the Future of NLP in Finance. 93–98. 10.61351/mf.v1i1.43

[B64] Zhang B. Hu Z. Wu P. Huang H. Xiang J. (2023). EPT: a data-driven transformer model for earthquake prediction. Eng. Appl. Artif. Intell. 123, 1–17. 10.1016/j.engappai.2023.106176

